# Reduced Risk of Parkinson's Disease in Users of Calcium Channel Blockers: A Meta-Analysis

**DOI:** 10.1155/2015/697404

**Published:** 2015-02-03

**Authors:** Kapil Gudala, Raju Kanukula, Dipika Bansal

**Affiliations:** Department of Pharmacy Practice, National Institute of Pharmaceutical Education and Research, SAS Nagar, Punjab 160062, India

## Abstract

*Aim*. To pool the data currently available to determine the association between calcium channel blockers (CCBs) and risk of Parkinson's disease (PD). *Methods*. Literature search in PubMed, EBSCO, and Cochrane library was undertaken through March 2014, looking for observational studies evaluating the association between CCBs use and PD. Pooled relative risk (RR) estimates and 95% confidence intervals (CIs) were calculated using random-effects model. Subgroup analyses, sensitivity Analysis, and cumulative meta-analysis were also performed. *Results*. Six studies were included in our meta-analysis according to the selection criteria, including three cohort studies and three case-control studies involving 27,67,990 subjects including 11,941 PD cases. We found CCBs use was associated with significant decreased risk of PD, compared with not using CCBs (random effects model pooled RR, 0.81 (95% CI, 0.69–0.95)); a significant heterogeneity was found between studies (*P* = 0.031; *I* ^2^ 54.6%). Both the classes of CCB, that is, dihydropyridine calcium channel blockers (DiCCB) (0.80 (95% CI, 0.65–0.98) *P* = 0.032) and non-DiCCB (0.70 (95% CI, 0.53–0.92) *P* = 0.013), were found to be reducing the risk of PD. *Conclusion*. In our analysis, we found that CCBs use was associated with a Significantly decreased risk of PD compared with non-CCB use.

## 1. Introduction

Approximately 1% of the population over 60 years of age suffers from Parkinson's disease (PD) which is a second most common chronic progressive neurodegenerative disorder in the elderly after Alzheimer's disease [1]. It has been characterized clinically by three motor symptoms, which includes resting tremors, rigidity, and bradykinesia [2]. Pathology involved in PD is the loss or degeneration of dopaminergic neurons in the substantia nigra of the midbrain and neuronal lewy bodies development. The results in the experimental therapies for treating PD were very limited [3]. A systematic review suggests that the centrally acting calcium channel blockers (CCBs) may have disease-modifying effects, and there were no drugs to prevent the disease or slow its progression [4].

Recent interest in antihypertensive drugs, especially CCBs, has been triggered by the belief that these medications, which inhibit nitric oxide, tumor necrosis factor-alpha, and interleukin-1 beta synthesis, thus reduce oxidative stress and the inflammatory response, which might be neuroprotective [5]. Experiments in animal models indicated that the voltage-gated calcium channel subtype Ca (V) 1.3 has a function in making neurons vulnerable to neurodegeneration [6].

Several observational studies have been conducted to examine the association between CCBs use and PD risk and have generated mixed results. Until now, no definite conclusion on this topic has been established. In the present meta-analysis, we examined the CCB use in relation to risk of PD.

## 2. Materials and Methods

### 2.1. Literature Search

Two authors independently performed the literature search by using MedLine (PubMed), EBSCO, and the Cochrane library databases up to March 2014. Search terms include “((((calcium channel blockers) OR antihypertensive agents) OR calcium antagonists) OR dihydropyridine calcium channel blockers) AND parkinson disease” with limits of humans and English language. Titles and abstracts of the resulting articles had been examined to exclude irrelevant studies. Full texts of remaining articles were read to extract information on the topic of interest. Bibliographies and citation sections of retrieved articles had been reviewed for additional pertinent studies.

### 2.2. Inclusion and Exclusion Criteria

The studies considered in this meta-analysis were all observational (cohort or case-control) studies that evaluated exposure to CCBs and risk of PD. Articles were excluded if they were reviews, letters to the editor without original data, editorials, case reports, or clinical trials. When there were multiple publications from the same population, only data from the most recent report were included in the meta-analysis and the remaining was excluded. Any discrepancies were addressed by a joint reevaluation of the original article.

### 2.3. Data Extraction

Two authors independently reviewed the primary studies to assess the appropriateness for inclusion in the present meta-analysis and data which has been extracted. The following information was extracted from each study: (i) first author's last name, year of publication, and country of the population studied; (ii) study design; (iii) number of subjects and number of PD cases; (iv) effect estimates and 95% confidence intervals (CIs); (v) assessment of CCB exposure; (vi) PD assessment; and (vii) control for confounding factors by matching or adjustments, if applicable. We extracted the effect estimates that reflected the greatest degree of control for potential confounder.

### 2.4. Quality Assessment

Two authors using the Newcastle-Ottawa Scale (NOS) [7] assessed the quality of each study independently. The NOS consists of three parameters of quality: selection, comparability, and outcome/exposure and it assigns a maximum of four points for selection, two points for comparability, and three points for exposure/outcome. Therefore, 9 points altogether reflect the high quality and 7-8 points reflect medium quality and six or less points reflect low quality. Any discrepancies were addressed by a joint revaluation of the original article with a third author.

### 2.5. Data Synthesis and Analysis

Because the risk of PD is low, the risk ratio (RR) in prospective cohort studies mathematically approximates the odds ratio [8], therefore permitting the combination of cohort and case-control studies. Publication bias was assessed using Egger's regression asymmetry test [9, 10]. To assess the heterogeneity among studies, we used the Cochran *Q* and *I* ^2^ statistics; for the *Q* statistic, a *P* value <0.10 and for *I* ^2^, a value >50% was considered statistically significant for heterogeneity [11]. The primary measure pooled RR of PD from individual studies, calculated using the random-effects model (DerSimonian and Laird method) [12, 13], which accounts for heterogeneity among studies. All analyses were performed using Comprehensive Meta-Analysis software version 2. All statistical tests were two-sided and *P* < 0.05 was considered statistically significant, except otherwise specified.

The primary outcome in this meta-analysis was reported as RR with 95% CI of developing PD in CCB users. Subgroup analyses were performed according to (i) dihydropyridine calcium channel blockers (DiCCBs) versus non-DiCCBs; (ii) individual type of CCB; (iii) dose; (iv) duration; (v) study design (cohort and case-control); (vi) gender; and (vii) age group to examine the impact of these factors on the association. To evaluate the stability of our results, we also performed a one-way sensitivity analysis. The present work was performed in this meta-analysis as per the guidelines for the meta-analysis PRISMA [14].

## 3. Results

### 3.1. Search Results

**Figure 1:**
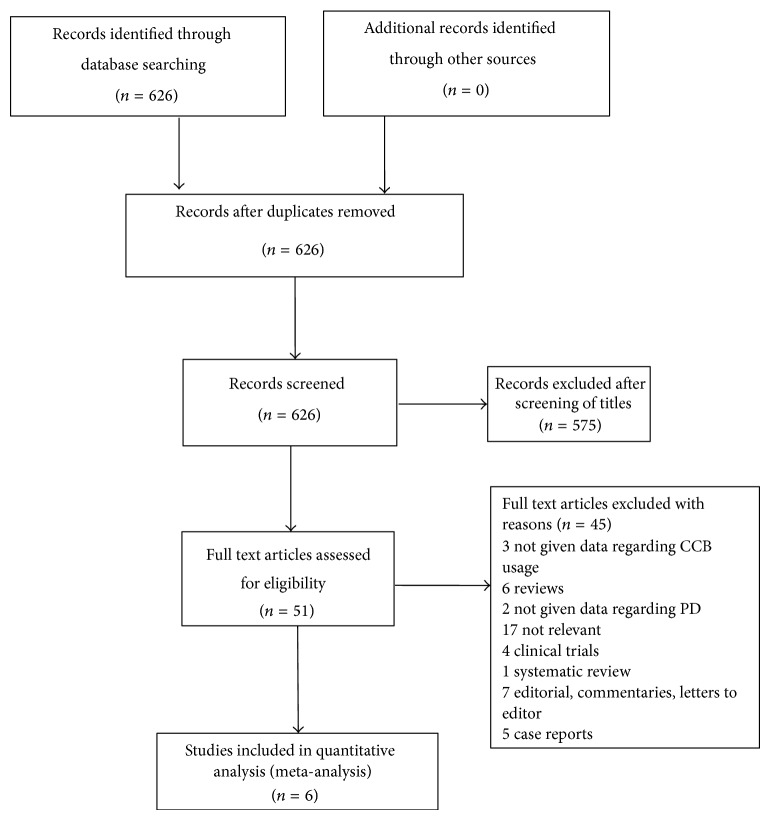
Flowchart representing the selection process.

A total of 626 articles were identified during the initial search (Figure 1). After screening the titles of 626 articles, 575 articles were excluded, as they were found irrelevant. Full text of 51 articles was collected and read. After detailed evaluation, 45 articles were found to be ineligible as there were reviews, editorials, case reports, and others which did not meet the inclusion criteria (Figure 1). A total of 6 studies were included for final analysis [15–20].

### 3.2. Study Characteristics

Six relevant studies were identified, including three cohort [15–17] and three case-control [18–20] studies involving a total of 27,67,990 subjects including 11,941 PD cases.

Three cohort studies involve [15–17] (Table 1) 27,48,578 participants with more than 2,06,000 CCB users out of which 6,182 were incident PD cases. Participants were followed up for 4 to 16 years and the studies have been published between 2009 and 2012. Pasternak et al. [15] study is a historical cohort study in being the biggest cohort among the three studies. Simon et al. [17] have done analysis by combining both the Nurses Health Study (NHS) and Health Professionals Follow-Up Study (HPFS). Louis et al. have reported the results of both cross-sectional and prospective analysis. However, present analysis has included only the prospective results of Louis et al. [16].

Three population-based case-control studies [18–20] (Table 2) involving 5,759 PD cases and 13,653 controls were published in between 2007 and 2010. All three studies are population-based studies, which assessed PD or CCB usage from national database or medical records or from pharmacy database.

### 3.3. Main Results

**Figure 2:**
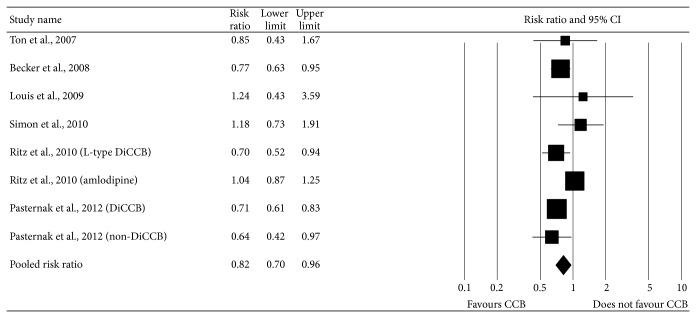
Combined estimate of risk ratio and 95% confidence intervals of Parkinson's disease associated with calcium channel blockers use based on six studies (three case-control and three cohort) of 27,67,990 subjects including 11,941 PD cases. Squares indicate RR in each study. The square size is proportional to the weight of the corresponding study in the meta-analysis; the length of horizontal lines represents the 95% CI. The shaded diamond indicates the combined RR and 95% CI (random-effects model).

As a significant heterogeneity was found (*P* = 0.031; *I* ^2^ 54.6%), random-effects model was chosen over a fixed effects model. We found CCB use was associated significantly with decreased risk of PD compared with not using CCB (pooled RR, 0.81 (95% CI, 0.69–0.96)). The multivariable adjusted RRs of use of CCB and risk of PD for each study and grouped data of all studies are shown in Figure 2. Visual examination of the funnel plot revealed minimal asymmetry (data not shown), further confirmed by Egger's test (*P* = 0.68) indicating little or no publication bias in our analysis.

### 3.4. Subgroup Analysis

Table 3 presents the results of subgroup analyses straitened by characteristics of study designs and populations. When cohort studies were analyzed alone [15–17], the pooled RR was found to be 0.73 (95% CI, 0.64–0.84). Using case-control studies alone [18–20], we found that the pooled RR was 0.84 (95% CI, 0.68–1.04). We found a significant difference between studies according to study design, where cohort studies significantly showed decreased risk of PD in CCB users. Although the RR of case-control studies is nonsignificant but still the effect estimate is on lower side.

Both the classes of CCB, that is, DiCCB (0.80 (95% CI, 0.65–0.98) *P* = 0.032) and non-DiCCB (0.70 (95% CI, 0.53–0.92) *P* = 0.013), were found to be reducing the risk of PD. We found a significant reduced risk of PD in females 0.67 (95% CI, 0.55–0.81) *P* = 0.243, in contrary to males 0.85 (95% CI, 0.66–1.12) *P* < 0.001.

To test the robustness of our findings, we also performed a sensitivity analysis. To do this, the overall effect size was calculated by removing one study at a time. This analysis showed no significance variation when excluding the Pasternak et al. [15] study 0.85 (95% CI, 0.71–1.01) *P* = 0.080 and Becker et al. [19] study 0.83 (95% CI, 0.68–1.01) *P* = 0.07.

Subgroup of studies having high quality [15, 19] presented significant inverse association (RR 0.70 95% CI, (0.61–0.81), *P* < 0.001) compared to studies having medium quality [16–18, 20] (RR 0.89 95% CI, (0.72–1.09) *P* = 0.272) (Table 3). Studies having better-quality scores (NOS score 9) showed a significant decreased risk of PD (0.70 95% CI, (0.61–0.81), *P* < 0.001).

## 4. Discussion

In the past decade, the role of CCBs in reduction of PD has been understood increasingly. With the present pooled analysis of 6 observational studies, a 19% reduction in PD risk among CCBs users as compared to nonusers was observed. CCBs are one of the most important antihypertensive drugs. The present analysis demonstrated the potential neuroprotective role of CCBs in reducing the risk of PD.

The etiopathogenesis of PD is complex. It involves *α*-synuclein deposition, dysfunction of protein turnover, and mitochondrial dysfunction leading to neuronal loss via excitotoxicity, calcium overload, and apoptosis [21]. Factors that potentiate pathological *α*-synuclein aggregation include posttranslational modifications, oxidative stress, and raised intracellular calcium ion [22, 23].* In vitro* culture models showed that transient increases of intracellular calcium induce cytoplasmic *α*-synuclein aggregates [23, 24]. In addition to the intracellular calcium overload, oxidative stress cooperatively promotes *α*-synuclein aggregation. By blocking the influx of calcium, CCBs can prevent or stop the progression of PD [23]. Dopaminergic neurons in substantia nigra possess L-type voltage gated calcium channels 1.3 for their pacemaker activity [25]. Kang et al. reported that, by selectively antagonizing Ca (V) 1.3 L-type channels, one could provide a solution for diminishing cell loss in PD with minimal side effects [26]. This provides a potential therapeutic target, by using drugs that modulate the amount of free Ca^+2^ in the cell. An array of CCBs are approved by USFDA to treat hypertension which could be tried to lessen the increase in intracellular Ca^+2^ seen in aged neurons in patients at high risk of developing PD based on family history.

Evidence on association between hypertension and risk of PD is conflicting. Prospective studies [27–29] showed an increased risk of PD in hypertensive patients. Some speculative mechanisms include untreated chronic hypertension which may lead to ischemic cerebrovascular lesions, increased oxidative stress, and modulation of central renin-angiotensin system (RAS) leading to PD [27]. It is not clear that whether lowering blood pressure has any role in reducing the risk of PD. The observed effect of decreased risk of PD in CCB users is assumed primarily by its neuroprotection action and not due to reduction of blood pressure in patients with hypertension.

Other antihypertensive drugs were also studied as potential agents to prevent or to stop progression of PD.* In vitro* and few* in vivo* studies have shown the role of agents modulating the RAS such as angiotensin converting enzyme inhibitors (ACEIs) and angiotensin receptor blockers (ARBs) [30]. Angiotensin type II when binds to the angiotensin type 1 receptor (AT1) activates the nicotinamide adenine dinucleotide phosphate oxidase complex, thus providing a major source of oxidative stress. In addition, activation of the AT_1_ receptor stimulates the NF-B signal transduction pathway which facilitates the synthesis of inflammatory mediators, which cause inflammation and later cell death. Thus ARBs and ACEIs act by modulating the oxidative stress and inflammation at the level of dopaminergic neurons in substansia nigra and basal ganglia. This makes them potential future targets to prevent or to stop progression of PD [30].

In our subgroup analysis, we found more pronounced reduced risk in women among CCBs users 0.67 (0.55–0.81, *P* = 0.243) as compared to men 0.85 (0.66–1.12, *P* < 0.001). These results are well coincided with the results published by Becker et al. [19]. Our study results were found to be significantly affected by study design, which might possibly be due to large sample size in cohort studies [15–17] as compared to case-control studies [18–20]. The definition of CCBs use in cohort and case-control studies is different. Moreover, none of the studies except study by Pasternak et al. [15] provided data regarding individual type of CCBs, duration, and dose. They emphasized on the protective association of amlodipine and felodipine with reduced risk of PD. They also concluded that individuals using high doses of amlodipine were at lower risk compared to those using standard dose. However, similar correlation was not observed in case of felodipine and nifedipine. Moreover, the results were nonconclusive because of low sample size.

Subgroup analysis revealed that use of nondihydropyridine CCBs was reported in only two studies [15, 19]. We found a significant reduced risk of PD in subgroup of nondihydropyridine CCBs users. The observed effect may explain that the protective role of CCBs in PD may not be limited to inhibition of voltage gated calcium channels [15].

A word of caution is necessary in interpretation of the analysis because sensitivity analysis by excluding study by Pasternak et al. [15] or Becker et al. [19] resulted in nonsignificant decrease in the RR of PD. The reason for this can be that these are two large well-conducted studies, which reported a decreased risk of PD in CCB users. These have a major contribution while pooling of effect estimates. Moreover subgroup analysis of high quality studies [15, 20] further suggested decreased risk of PD in CCB users, which further helps to conclude the usefulness of CCBs use in prevention of PD.

Several limitations of our study need to be addressed. Our analysis was restricted to articles in English language, which may have led to somewhat biased results. All the studies included were observational studies with different follow-up periods, and no standard definition of CCBs usage was there. The specific role of individual drugs and doses was also not possible because of nonreporting in the studies.

Our analysis suggests that CCBs may have protective role in PD. However, future prospective studies with larger sample size are required to understand the effect of individual CCBs at various dose and duration of use.

## Figures and Tables

**Table 1:** Characteristics of cohort studies included in meta-analysis.

Author, year (country)^a^	Cohort name	Cohort size	Follow-up period (start–end year)	Assessment of CCB use	Number of CCB users	Assessment of PD	Number of PD cases	Quality rating (NOC)
Pasternak et al. [15] 2012 (Denmark)^a^	NR	25,73,281	8 (1998–2006)	Prescription drug registry	2,02,836	National patient registry	5,711	9^b^

Simon et al. [17] 2010 (USA)^a^	Nurses Health study & Health Professionals Follow-Up Study	1,71,355	16 (1986–2002)	Self-reported through structured questionnaire	3,826	Self-reported and after confirmed by medical records and physician	421	7^c^

Louis et al. [16] 2009 (Spain)^a^	Neurological Disorder in Central Spain	3,942	4 (1994–1998)	Self-reported	NR	Presence of any two cardinal signs and physician confirmed PD	NR	8^c^

^a^Country of study conducted.

^b^High quality.

^c^Medium quality.

USA: United States of America, NR: not reported, CCB: calcium channel blockers, PD: Parkinson's disease, and NOC: Newcastle-Ottawa Scale.

**Table 2:** Characteristics of case-control studies included in meta-analysis.

Author, year (country)^a^	Period of recruitment	Source of study population	Study size	Number of PD patients	Assessment of CCB usage	Assessment of PD	Quality rating (NOC)
Ritz et al. [18] 2010 (Denmark)	2001–2006	Population based	11,582	1,931	National pharmacy database	Hospital register	8^c^

Becker et al. [19] 2008 (UK)	1994–2005	Population based	7,274	3,637	General practice research database	General practice research database	8^c^

Ton et al. [20] 2007 (USA)	1992–2002	Population based	556	191	Medical records	Medical records and cardinal signs	9^b^

^a^Country of study conducted.

^b^High quality.

^c^Medium quality.

UK: United Kingdom, USA: United States of America, CCB: calcium channel blockers, PD: Parkinson's disease, and NOC: Newcastle-Ottawa Scale.

**Table 3:** Overall effect estimates for Parkinson's disease and calcium channel blockers use according to study characteristics.

Characteristic	*n*	Risk ratio (95% CI)	*P* value	Heterogeneity	
				*I* ^2^ (%)	Cochrane *Q*
All studies	6	0.81 (0.69–0.96)	0.014^a^	54.6	0.031
Study design					
Cohort	3	0.73 (0.64–0.84)	<0.001^a^	42.6	0.156
Case-control	3	0.84 (0.68–1.04)	0.111	58.1	0.06
Class of CCB					
DiCCB	4	0.80 (0.65–0.98)	0.032^a^	72.9	0.011
Non-DiCCB	2	0.70 (0.53–0.92)	0.013^a^	0	0.546
Gender					
Men	3	0.85 (0.66–1.12)	0.243	53.1	0.118
Women	3	0.67 (0.55–0.81)	<0.001^a^	0	0.919
Sensitivity analysis					
All studies except Pasternak et al. [15]	—	0.85 (0.71–1.01)	0.080^a^	NA	NA
All studies except Becker et al. [19]	—	0.83 (0.68–1.01)	0.071^a^	NA	NA
Quality					
High	2	0.70 (0.61–0.81)	<0.001^a^	0	0.774
Medium	4	0.89 (0.72–1.09)	0.272	55.3	0.062

^a^ *P *value representing significant inverse association between CCBs use and Parkinson's disease.

CCB: calcium channel blockers, DiCCB: dihydropyridine calcium channel blockers, and NA: not available.

CI: confidence interval.
